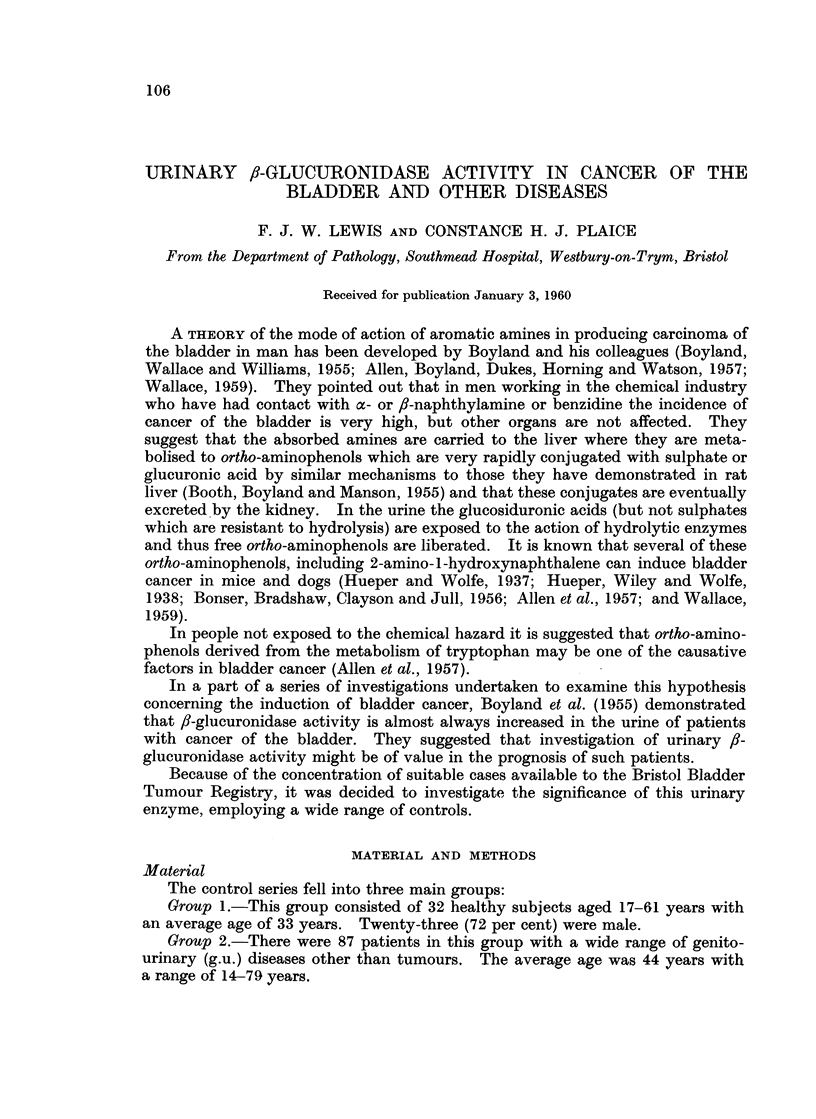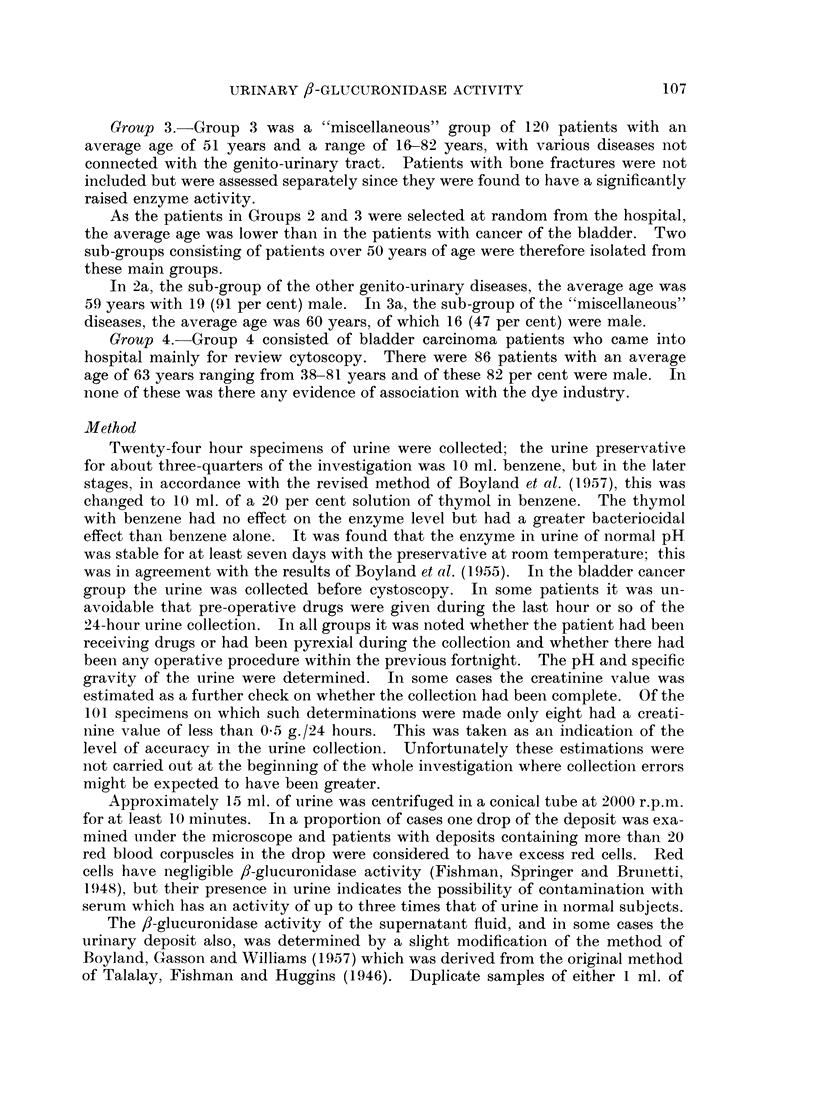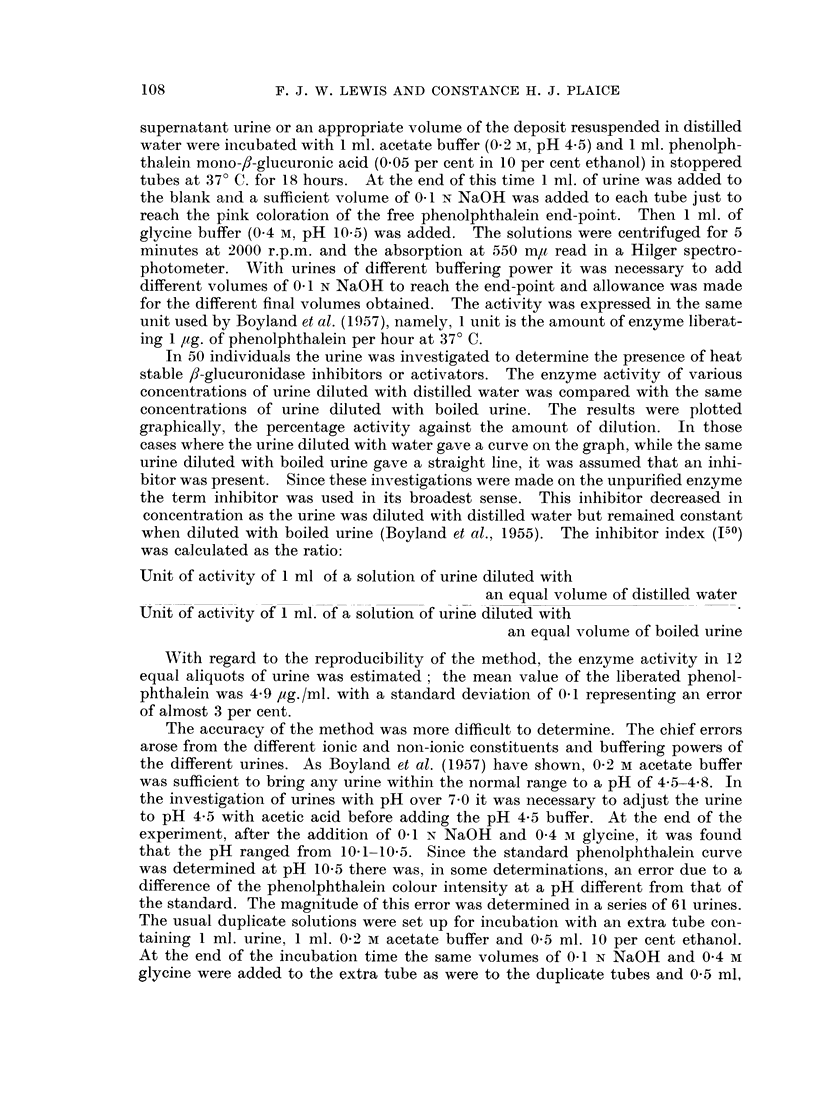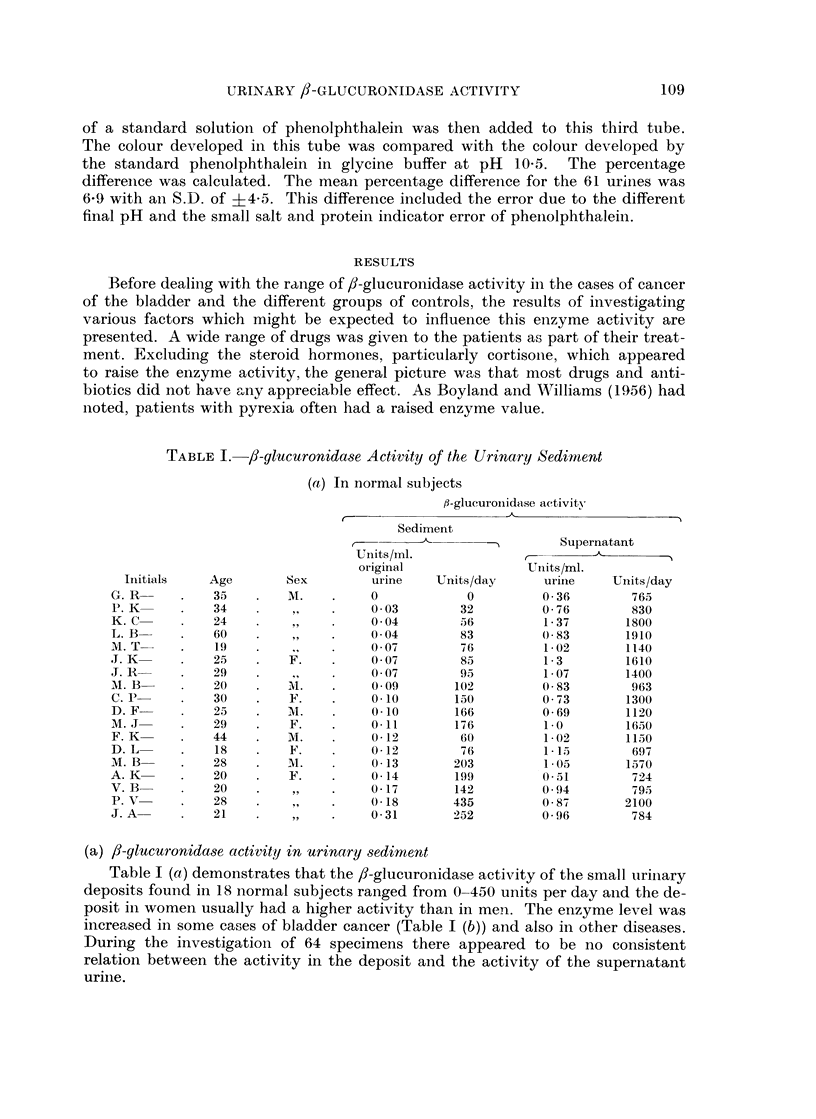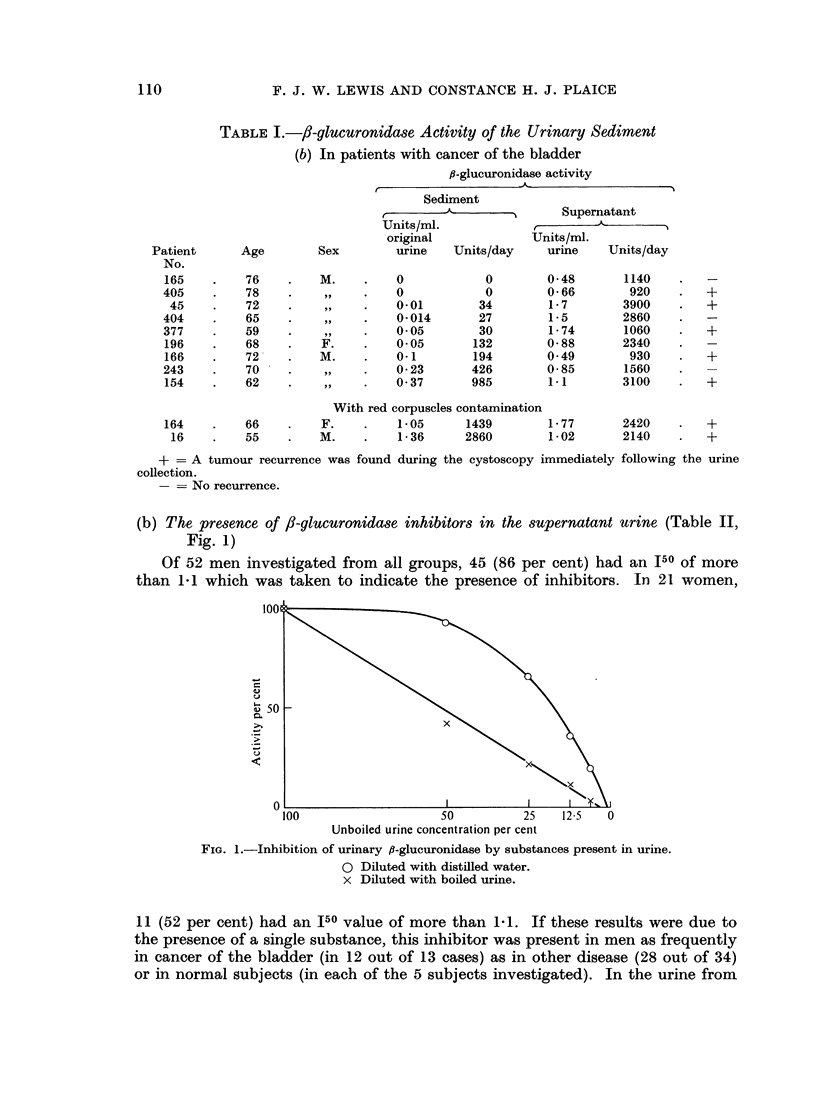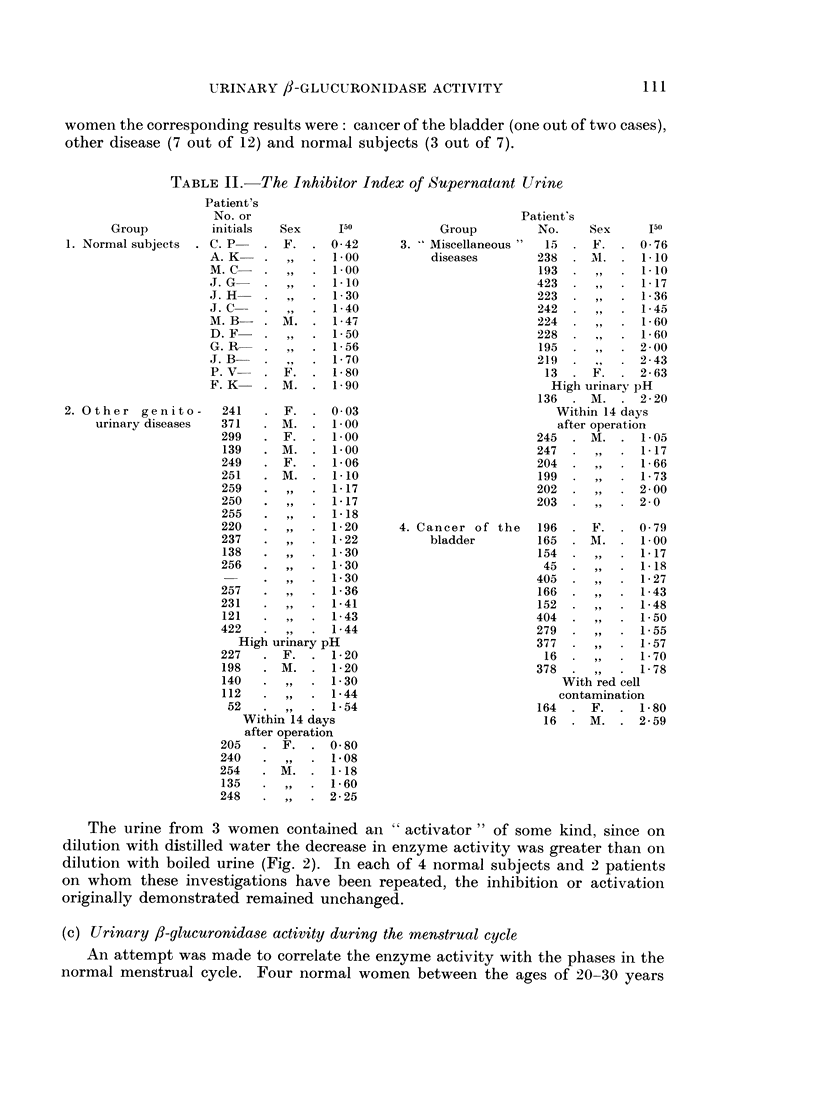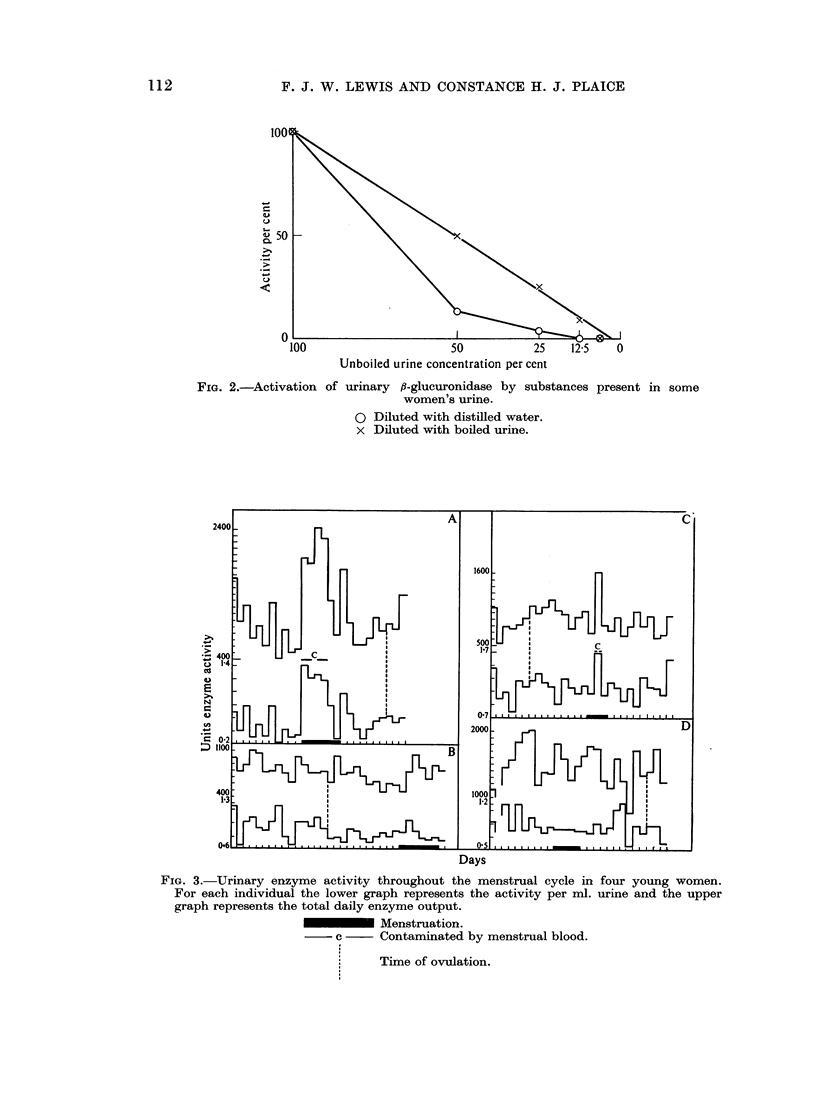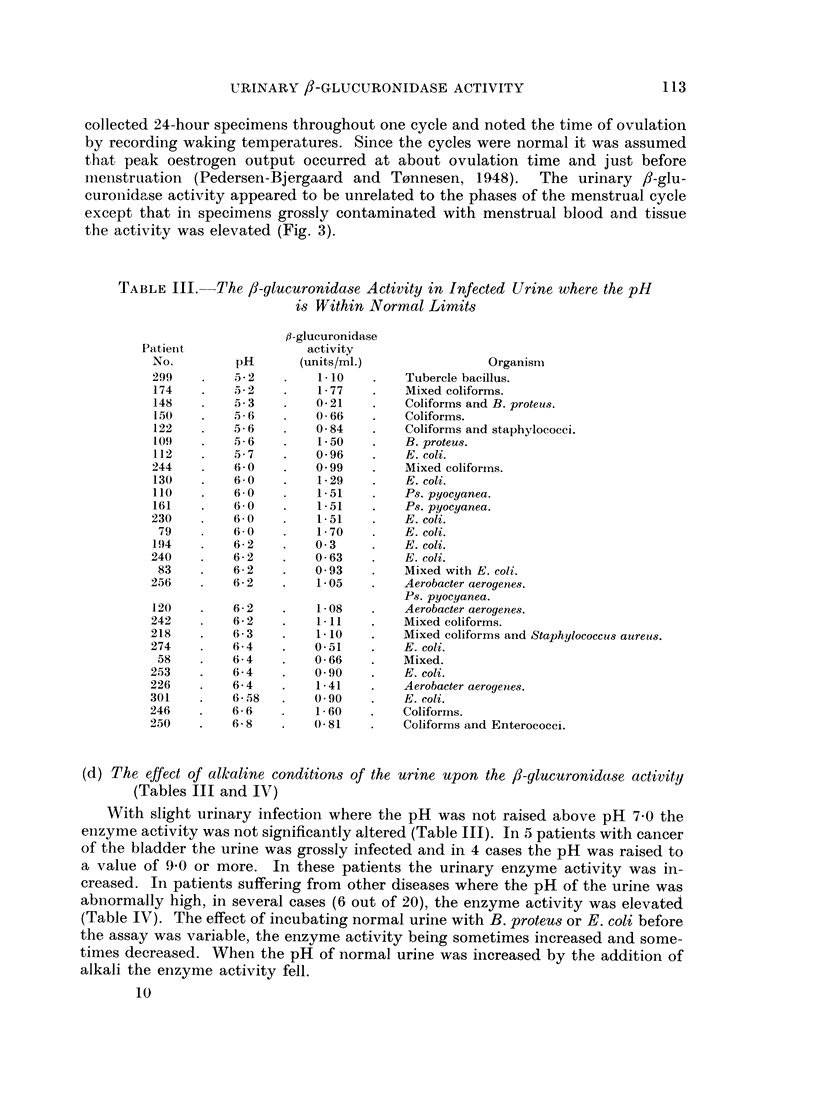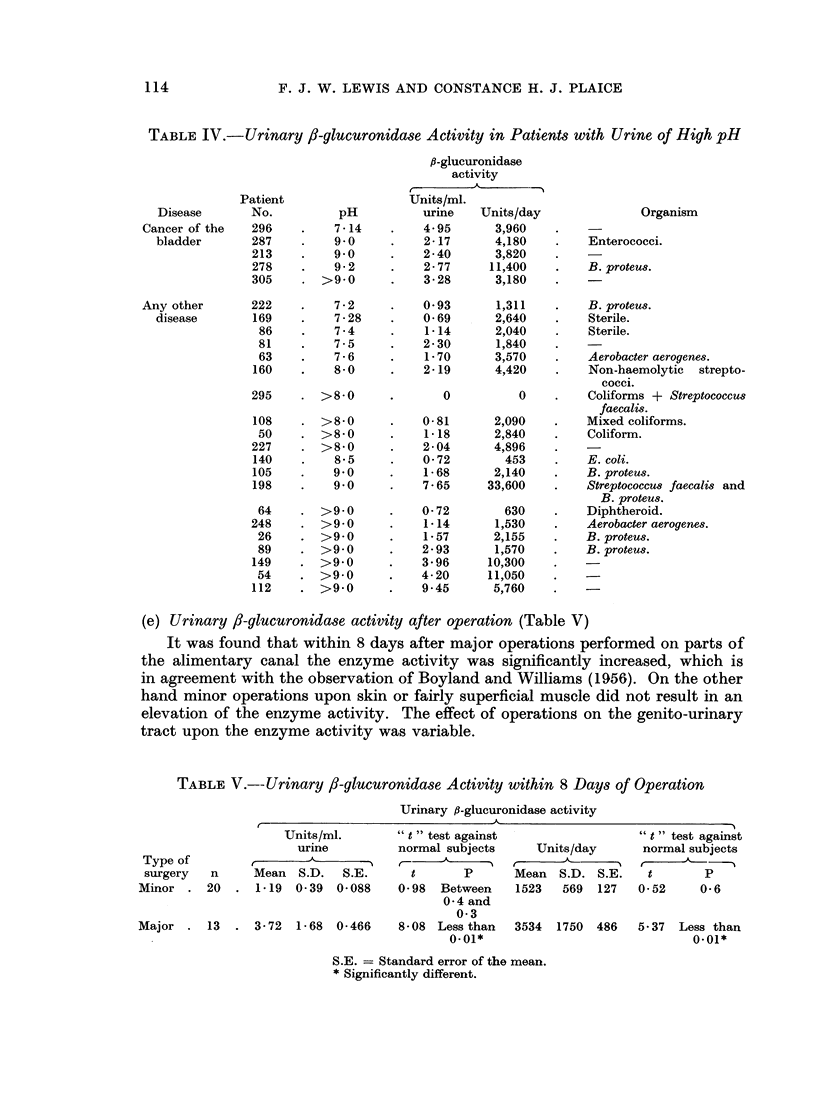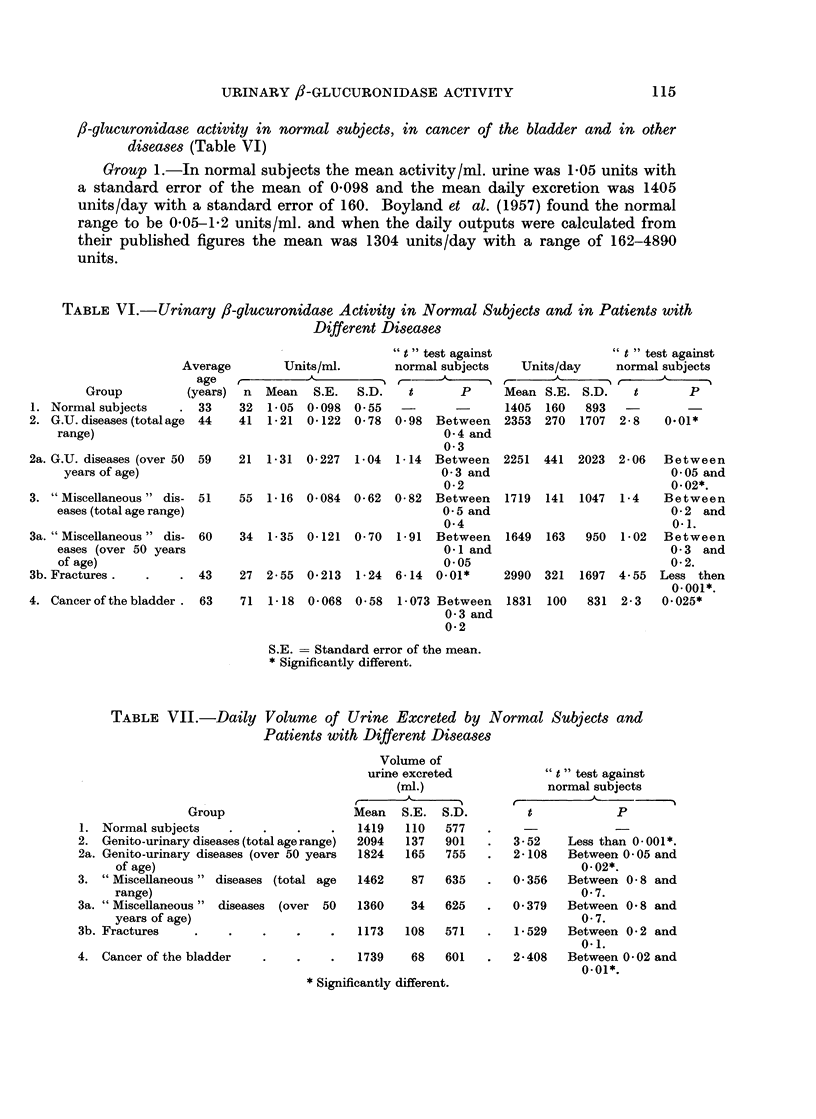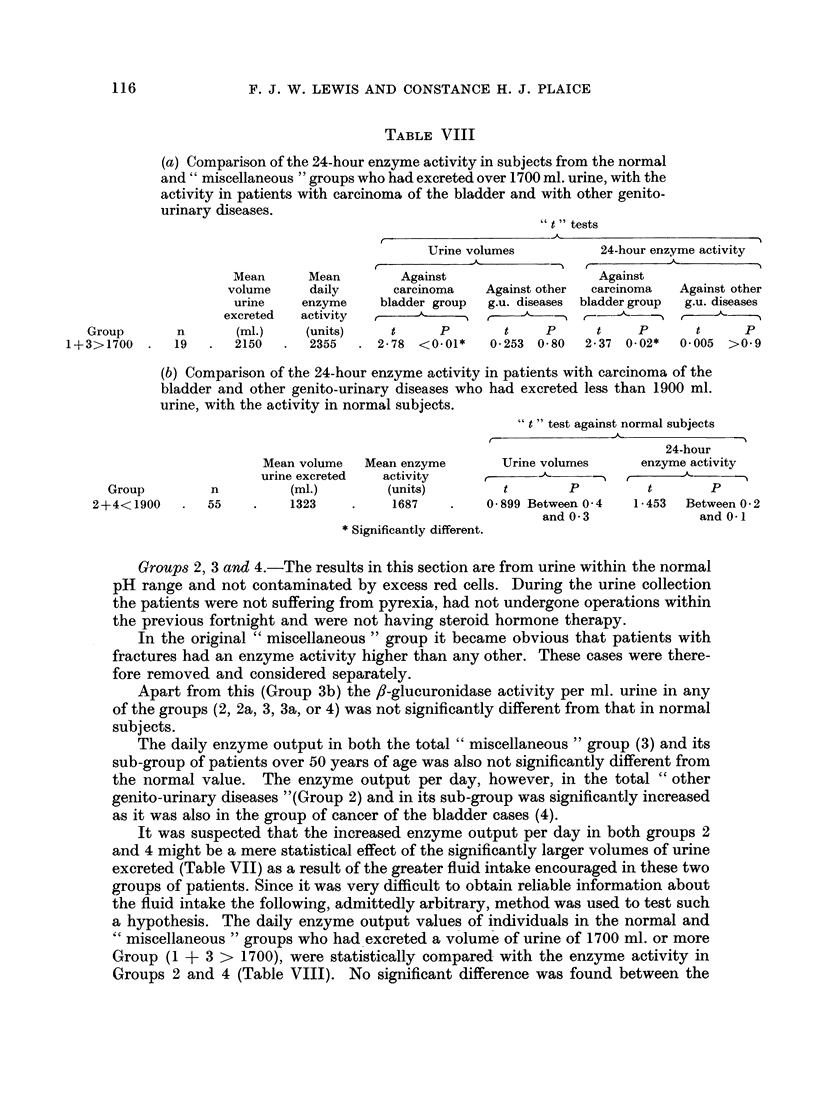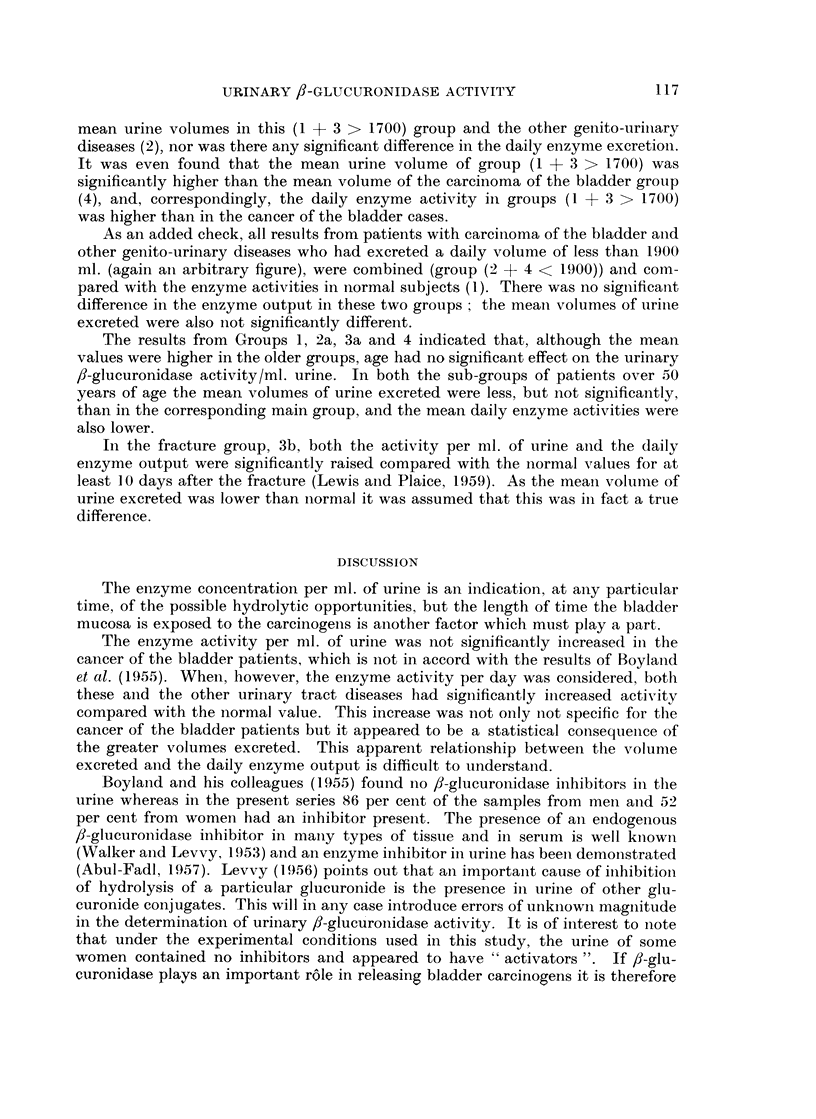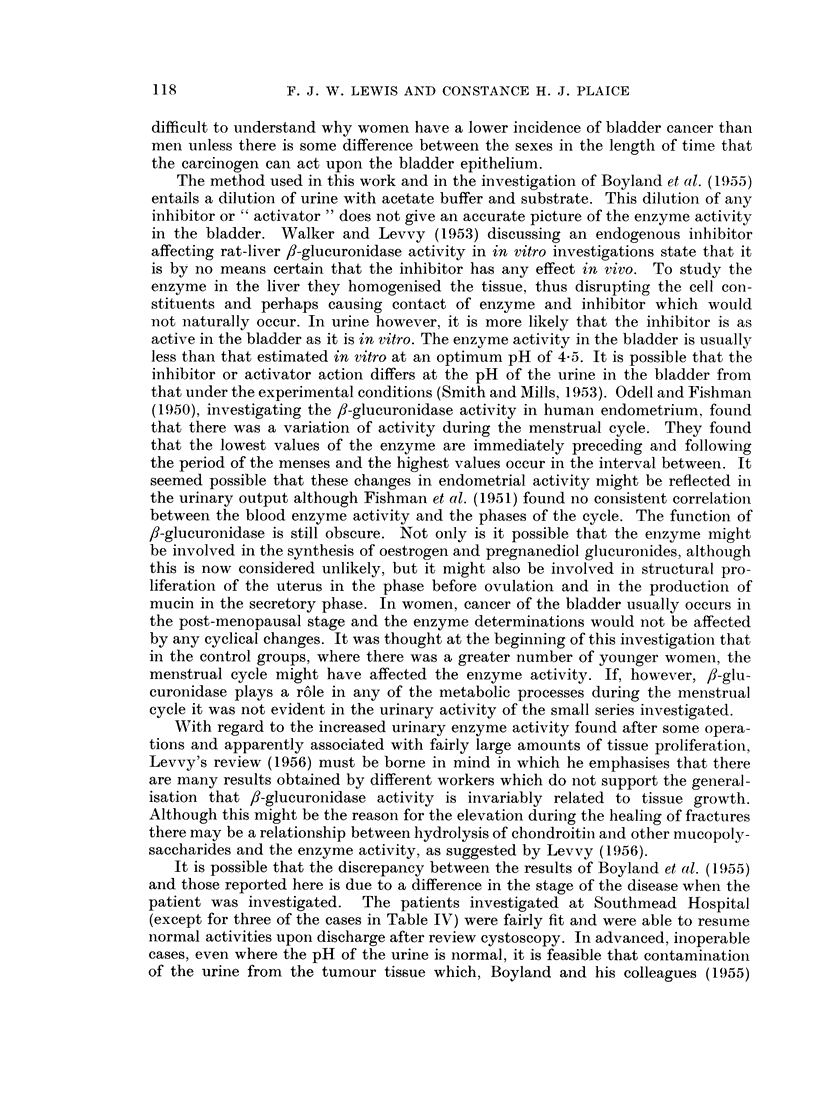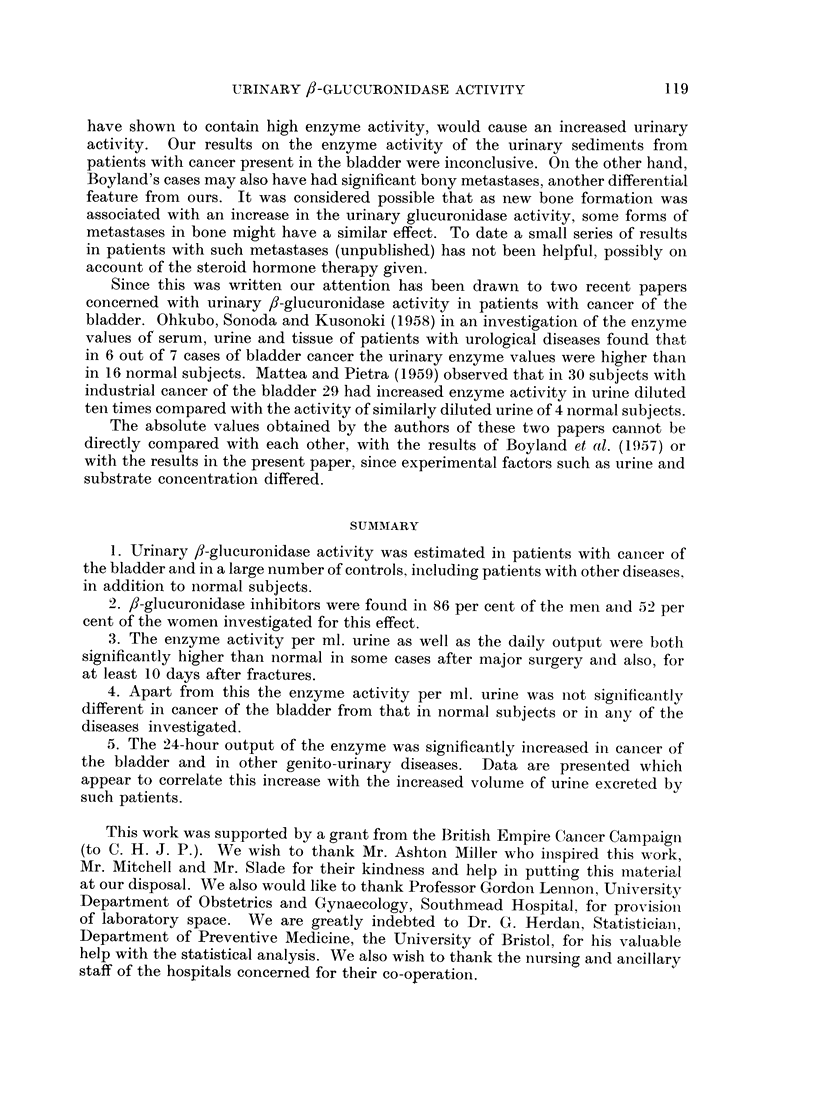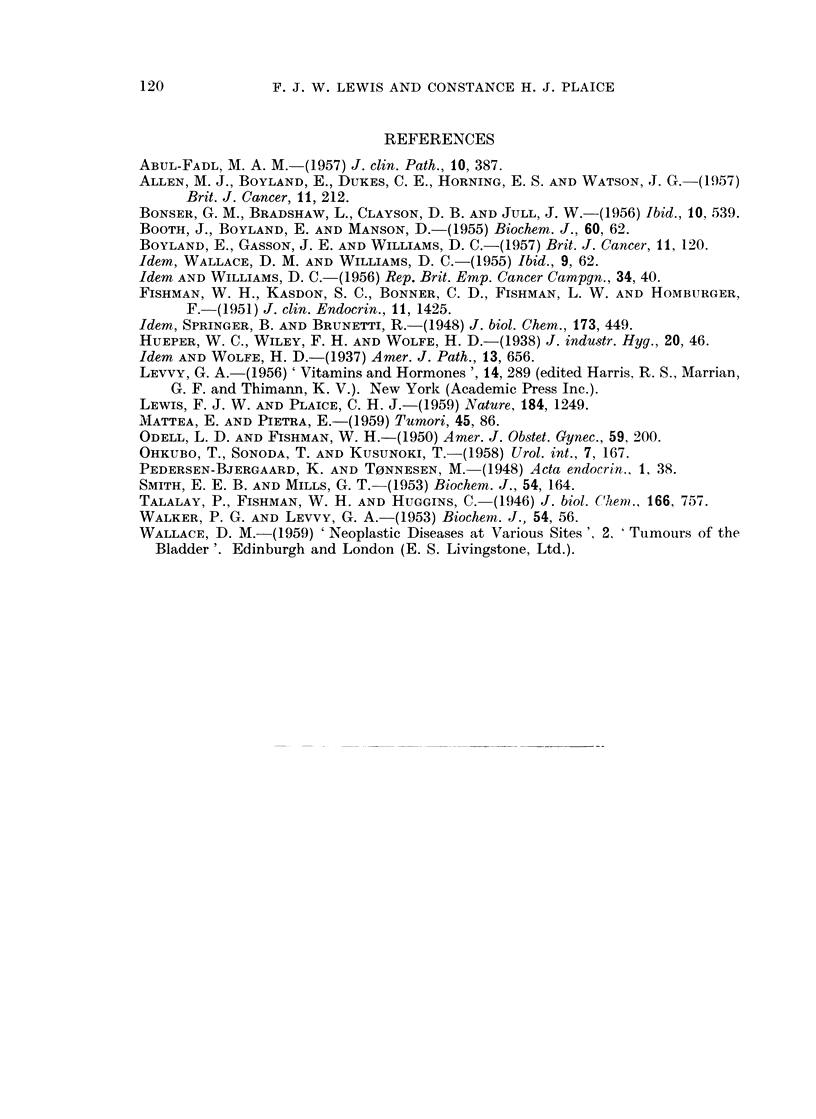# Urinary β-Glucuronidase Activity in Cancer of the Bladder and other Diseases

**DOI:** 10.1038/bjc.1960.13

**Published:** 1960-03

**Authors:** F. J. W. Lewis, Constance H. J. Plaice


### URINARY β-GLUCURONIDASE ACTIVITY IN CANCER OF THE BLADDER AND OTHER DISEASES

F. J. W. LEWIS AND CONSTANCE H. J. PLAICE

*From the Department of Pathology, Southmead Hospital, Westbury-on-Trym, Bristol*

Received for publication January 3, 1960

A THEORY of the mode of action of aromatic amines in producing carcinoma of the bladder in man has been developed by Boyland and his colleagues (Boyland, Wallace and Williams, 1955; Allen, Boyland, Dukes, Horning and Watson, 1957; Wallace, 1959). They pointed out that in men working in the chemical industry who have had contact with α- or β-naphthylamine or benzidine the incidence of cancer of the bladder is very high, but other organs are not affected. They suggest that the absorbed amines are carried to the liver where they are metabolised to *ortho*-aminophenols which are very rapidly conjugated with sulphate or glucuronic acid by similar mechanisms to those they have demonstrated in rat liver (Booth, Boyland and Manson, 1955) and that these conjugates are eventually excreted by the kidney. In the urine the glucosiduronic acids (but not sulphates which are resistant to hydrolysis) are exposed to the action of hydrolytic enzymes and thus free *ortho*-aminophenols are liberated. It is known that several of these *ortho*-aminophenols, including 2-amino-1-hydroxynaphthalene can induce bladder cancer in mice and dogs (Hueper and Wolfe, 1937; Hueper, Wiley and Wolfe, 1938; Bonser, Bradshaw, Clayson and Jull, 1956; Allen *et al.*, 1957; and Wallace, 1959).

In people not exposed to the chemical hazard it is suggested that *ortho*-aminophenols derived from the metabolism of tryptophan may be one of the causative factors in bladder cancer (Allen *et al.*, 1957).

In a part of a series of investigations undertaken to examine this hypothesis concerning the induction of bladder cancer, Boyland *et al.* (1955) demonstrated that β-glucuronidase activity is almost always increased in the urine of patients with cancer of the bladder. They suggested that investigation of urinary β-glucuronidase activity might be of value in the prognosis of such patients.

Because of the concentration of suitable cases available to the Bristol Bladder Tumour Registry, it was decided to investigate the significance of this urinary enzyme, employing a wide range of controls.

### MATERIAL AND METHODS

### *Material*

The control series fell into three main groups:

*Group* 1.—This group consisted of 32 healthy subjects aged 17–61 years with an average age of 33 years. Twenty-three (72 per cent) were male.

*Group* 2.—There were 87 patients in this group with a wide range of genito-urinary (g.u.) diseases other than tumours. The average age was 44 years with a range of 14–79 years.

*Group* 3.—Group 3 was a "miscellaneous" group of 120 patients with an average age of 51 years and a range of 16–82 years, with various diseases not connected with the genito-urinary tract. Patients with bone fractures were not included but were assessed separately since they were found to have a significantly raised enzyme activity.

As the patients in Groups 2 and 3 were selected at random from the hospital, the average age was lower than in the patients with cancer of the bladder. Two sub-groups consisting of patients over 50 years of age were therefore isolated from these main groups.

In 2a, the sub-group of the other genito-urinary diseases, the average age was 59 years with 19 (91 per cent) male. In 3a, the sub-group of the "miscellaneous" diseases, the average age was 60 years, of which 16 (47 per cent) were male.

*Group* 4.—Group 4 consisted of bladder carcinoma patients who came into hospital mainly for review cytoscopy. There were 86 patients with an average age of 63 years ranging from 38–81 years and of these 82 per cent were male. In none of these was there any evidence of association with the dye industry.

*Method*

Twenty-four hour specimens of urine were collected; the urine preservative for about three-quarters of the investigation was 10 ml. benzene, but in the later stages, in accordance with the revised method of Boyland *et al.* (1957), this was changed to 10 ml. of a 20 per cent solution of thymol in benzene. The thymol with benzene had no effect on the enzyme level but had a greater bacteriocidal effect than benzene alone. It was found that the enzyme in urine of normal pH was stable for at least seven days with the preservative at room temperature; this was in agreement with the results of Boyland *et al.* (1955). In the bladder cancer group the urine was collected before cystoscopy. In some patients it was unavoidable that pre-operative drugs were given during the last hour or so of the 24-hour urine collection. In all groups it was noted whether the patient had been receiving drugs or had been pyrexial during the collection and whether there had been any operative procedure within the previous fortnight. The pH and specific gravity of the urine were determined. In some cases the creatinine value was estimated as a further check on whether the collection had been complete. Of the 101 specimens on which such determinations were made only eight had a creatinine value of less than 0·5 g./24 hours. This was taken as an indication of the level of accuracy in the urine collection. Unfortunately these estimations were not carried out at the beginning of the whole investigation where collection errors might be expected to have been greater.

Approximately 15 ml. of urine was centrifuged in a conical tube at 2000 r.p.m. for at least 10 minutes. In a proportion of cases one drop of the deposit was examined under the microscope and patients with deposits containing more than 20 red blood corpuscles in the drop were considered to have excess red cells. Red cells have negligible $\beta$-glucuronidase activity (Fishman, Springer and Brunetti, 1948), but their presence in urine indicates the possibility of contamination with serum which has an activity of up to three times that of urine in normal subjects.

The $\beta$-glucuronidase activity of the supernatant fluid, and in some cases the urinary deposit also, was determined by a slight modification of the method of Boyland, Gasson and Williams (1957) which was derived from the original method of Talalay, Fishman and Huggins (1946). Duplicate samples of either 1 ml. of supernatant urine or an appropriate volume of the deposit resuspended in distilled water were incubated with 1 ml. acetate buffer (0·2 M, pH 4·5) and 1 ml. phenolphthalein mono-$\beta$-glucuronic acid (0·05 per cent in 10 per cent ethanol) in stoppered tubes at 37° C. for 18 hours. At the end of this time 1 ml. of urine was added to the blank and a sufficient volume of 0·1 N NaOH was added to each tube just to reach the pink coloration of the free phenolphthalein end-point. Then 1 ml. of glycine buffer (0·4 M, pH 10·5) was added. The solutions were centrifuged for 5 minutes at 2000 r.p.m. and the absorption at 550 m$\mu$ read in a Hilger spectrophotometer. With urines of different buffering power it was necessary to add different volumes of 0·1 N NaOH to reach the end-point and allowance was made for the different final volumes obtained. The activity was expressed in the same unit used by Boyland *et al.* (1957), namely, 1 unit is the amount of enzyme liberating 1 $\mu$g. of phenolphthalein per hour at 37° C.

In 50 individuals the urine was investigated to determine the presence of heat stable $\beta$-glucuronidase inhibitors or activators. The enzyme activity of various concentrations of urine diluted with distilled water was compared with the same concentrations of urine diluted with boiled urine. The results were plotted graphically, the percentage activity against the amount of dilution. In those cases where the urine diluted with water gave a curve on the graph, while the same urine diluted with boiled urine gave a straight line, it was assumed that an inhibitor was present. Since these investigations were made on the unpurified enzyme the term inhibitor was used in its broadest sense. This inhibitor decreased in concentration as the urine was diluted with distilled water but remained constant when diluted with boiled urine (Boyland *et al.*, 1955). The inhibitor index ($I^{50}$) was calculated as the ratio:

$$\frac{\text{Unit of activity of 1 ml of a solution of urine diluted with an equal volume of distilled water}}{\text{Unit of activity of 1 ml. of a solution of urine diluted with an equal volume of boiled urine}}.$$

With regard to the reproducibility of the method, the enzyme activity in 12 equal aliquots of urine was estimated ; the mean value of the liberated phenolphthalein was 4·9 $\mu$g./ml. with a standard deviation of 0·1 representing an error of almost 3 per cent.

The accuracy of the method was more difficult to determine. The chief errors arose from the different ionic and non-ionic constituents and buffering powers of the different urines. As Boyland *et al.* (1957) have shown, 0·2 M acetate buffer was sufficient to bring any urine within the normal range to a pH of 4·5–4·8. In the investigation of urines with pH over 7·0 it was necessary to adjust the urine to pH 4·5 with acetic acid before adding the pH 4·5 buffer. At the end of the experiment, after the addition of 0·1 N NaOH and 0·4 M glycine, it was found that the pH ranged from 10·1–10·5. Since the standard phenolphthalein curve was determined at pH 10·5 there was, in some determinations, an error due to a difference of the phenolphthalein colour intensity at a pH different from that of the standard. The magnitude of this error was determined in a series of 61 urines. The usual duplicate solutions were set up for incubation with an extra tube containing 1 ml. urine, 1 ml. 0·2 M acetate buffer and 0·5 ml. 10 per cent ethanol. At the end of the incubation time the same volumes of 0·1 N NaOH and 0·4 M glycine were added to the extra tube as were to the duplicate tubes and 0·5 ml.

of a standard solution of phenolphthalein was then added to this third tube. The colour developed in this tube was compared with the colour developed by the standard phenolphthalein in glycine buffer at pH 10·5. The percentage difference was calculated. The mean percentage difference for the 61 urines was 6·9 with an S.D. of $\pm 4 \cdot 5$. This difference included the error due to the different final pH and the small salt and protein indicator error of phenolphthalein.

### RESULTS

Before dealing with the range of β-glucuronidase activity in the cases of cancer of the bladder and the different groups of controls, the results of investigating various factors which might be expected to influence this enzyme activity are presented. A wide range of drugs was given to the patients as part of their treatment. Excluding the steroid hormones, particularly cortisone, which appeared to raise the enzyme activity, the general picture was that most drugs and antibiotics did not have any appreciable effect. As Boyland and Williams (1956) had noted, patients with pyrexia often had a raised enzyme value.

TABLE I.—*β-glucuronidase Activity of the Urinary Sediment*

(*a*) In normal subjects

| | | | β-glucuronidase activity | | | |
|---|---|---|---|---|---|---|
| | | | Sediment | | Supernatant | |
| Initials | Age | Sex | Units/ml. original urine | Units/day | Units/ml. urine | Units/day |
| G. R— | 35 | M. | 0 | 0 | 0·36 | 765 |
| P. K— | 34 | ,, | 0·03 | 32 | 0·76 | 830 |
| K. C— | 24 | ,, | 0·04 | 56 | 1·37 | 1800 |
| L. B— | 60 | ,, | 0·04 | 83 | 0·83 | 1910 |
| M. T— | 19 | ,, | 0·07 | 76 | 1·02 | 1140 |
| J. K— | 25 | F. | 0·07 | 85 | 1·3 | 1610 |
| J. R— | 29 | ,, | 0·07 | 95 | 1·07 | 1400 |
| M. B— | 20 | M. | 0·09 | 102 | 0·83 | 963 |
| C. P— | 30 | F. | 0·10 | 150 | 0·73 | 1300 |
| D. F— | 25 | M. | 0·10 | 166 | 0·69 | 1120 |
| M. J— | 29 | F. | 0·11 | 176 | 1·0 | 1650 |
| F. K— | 44 | M. | 0·12 | 60 | 1·02 | 1150 |
| D. L— | 18 | F. | 0·12 | 76 | 1·15 | 697 |
| M. B— | 28 | M. | 0·13 | 203 | 1·05 | 1570 |
| A. K— | 20 | F. | 0·14 | 199 | 0·51 | 724 |
| V. B— | 20 | ,, | 0·17 | 142 | 0·94 | 795 |
| P. V— | 28 | ,, | 0·18 | 435 | 0·87 | 2100 |
| J. A— | 21 | ,, | 0·31 | 252 | 0·96 | 784 |

### (a) *β-glucuronidase activity in urinary sediment*

Table I (*a*) demonstrates that the β-glucuronidase activity of the small urinary deposits found in 18 normal subjects ranged from 0–450 units per day and the deposit in women usually had a higher activity than in men. The enzyme level was increased in some cases of bladder cancer (Table I (*b*)) and also in other diseases. During the investigation of 64 specimens there appeared to be no consistent relation between the activity in the deposit and the activity of the supernatant urine.

TABLE I.—*β-glucuronidase Activity of the Urinary Sediment*

(*b*) In patients with cancer of the bladder

| Patient No. | Age | Sex | β-glucuronidase activity: Sediment, Units/ml. original urine | Sediment, Units/day | Supernatant, Units/ml. urine | Supernatant, Units/day | |
|---|---|---|---|---|---|---|---|
| 165 | 76 | M. | 0 | 0 | 0·48 | 1140 | — |
| 405 | 78 | „ | 0 | 0 | 0·66 | 920 | + |
| 45 | 72 | „ | 0·01 | 34 | 1·7 | 3900 | + |
| 404 | 65 | „ | 0·014 | 27 | 1·5 | 2860 | — |
| 377 | 59 | „ | 0·05 | 30 | 1·74 | 1060 | + |
| 196 | 68 | F. | 0·05 | 132 | 0·88 | 2340 | — |
| 166 | 72 | M. | 0·1 | 194 | 0·49 | 930 | + |
| 243 | 70 | „ | 0·23 | 426 | 0·85 | 1560 | — |
| 154 | 62 | „ | 0·37 | 985 | 1·1 | 3100 | + |
| With red corpuscles contamination | | | | | | | |
| 164 | 66 | F. | 1·05 | 1439 | 1·77 | 2420 | + |
| 16 | 55 | M. | 1·36 | 2860 | 1·02 | 2140 | + |

\+ = A tumour recurrence was found during the cystoscopy immediately following the urine collection.

— = No recurrence.

(b) *The presence of β-glucuronidase inhibitors in the supernatant urine* (Table II, Fig. 1)

Of 52 men investigated from all groups, 45 (86 per cent) had an $I^{50}$ of more than 1·1 which was taken to indicate the presence of inhibitors. In 21 women,

FIG. 1.—Inhibition of urinary β-glucuronidase by substances present in urine.

○ Diluted with distilled water.

× Diluted with boiled urine.

11 (52 per cent) had an $I^{50}$ value of more than 1·1. If these results were due to the presence of a single substance, this inhibitor was present in men as frequently in cancer of the bladder (in 12 out of 13 cases) as in other disease (28 out of 34) or in normal subjects (in each of the 5 subjects investigated). In the urine from women the corresponding results were : cancer of the bladder (one out of two cases), other disease (7 out of 12) and normal subjects (3 out of 7).

TABLE II.—*The Inhibitor Index of Supernatant Urine*

| Group | Patient's No. or initials | Sex | $I^{50}$ |
|---|---|---|---|
| 1. Normal subjects | C. P— | F. | 0·42 |
| | A. K— | ,, | 1·00 |
| | M. C— | ,, | 1·00 |
| | J. G— | ,, | 1·10 |
| | J. H— | ,, | 1·30 |
| | J. C— | ,, | 1·40 |
| | M. B— | M. | 1·47 |
| | D. F— | ,, | 1·50 |
| | G. R— | ,, | 1·56 |
| | J. B— | ,, | 1·70 |
| | P. V— | F. | 1·80 |
| | F. K— | M. | 1·90 |
| 2. Other genito-urinary diseases | 241 | F. | 0·03 |
| | 371 | M. | 1·00 |
| | 299 | F. | 1·00 |
| | 139 | M. | 1·00 |
| | 249 | F. | 1·06 |
| | 251 | M. | 1·10 |
| | 259 | ,, | 1·17 |
| | 250 | ,, | 1·17 |
| | 255 | ,, | 1·18 |
| | 220 | ,, | 1·20 |
| | 237 | ,, | 1·22 |
| | 138 | ,, | 1·30 |
| | 256 | ,, | 1·30 |
| | — | ,, | 1·30 |
| | 257 | ,, | 1·36 |
| | 231 | ,, | 1·41 |
| | 121 | ,, | 1·43 |
| | 422 | ,, | 1·44 |
| | High urinary pH | | |
| | 227 | F. | 1·20 |
| | 198 | M. | 1·20 |
| | 140 | ,, | 1·30 |
| | 112 | ,, | 1·44 |
| | 52 | ,, | 1·54 |
| | Within 14 days after operation | | |
| | 205 | F. | 0·80 |
| | 240 | ,, | 1·08 |
| | 254 | M. | 1·18 |
| | 135 | ,, | 1·60 |
| | 248 | ,, | 2·25 |

| Group | Patient's No. | Sex | $I^{50}$ |
|---|---|---|---|
| 3. " Miscellaneous " diseases | 15 | F. | 0·76 |
| | 238 | M. | 1·10 |
| | 193 | ,, | 1·10 |
| | 423 | ,, | 1·17 |
| | 223 | ,, | 1·36 |
| | 242 | ,, | 1·45 |
| | 224 | ,, | 1·60 |
| | 228 | ,, | 1·60 |
| | 195 | ,, | 2·00 |
| | 219 | ,, | 2·43 |
| | 13 | F. | 2·63 |
| | High urinary pH | | |
| | 136 | M. | 2·20 |
| | Within 14 days after operation | | |
| | 245 | M. | 1·05 |
| | 247 | ,, | 1·17 |
| | 204 | ,, | 1·66 |
| | 199 | ,, | 1·73 |
| | 202 | ,, | 2·00 |
| | 203 | ,, | 2·0 |
| 4. Cancer of the bladder | 196 | F. | 0·79 |
| | 165 | M. | 1·00 |
| | 154 | ,, | 1·17 |
| | 45 | ,, | 1·18 |
| | 405 | ,, | 1·27 |
| | 166 | ,, | 1·43 |
| | 152 | ,, | 1·48 |
| | 404 | ,, | 1·50 |
| | 279 | ,, | 1·55 |
| | 377 | ,, | 1·57 |
| | 16 | ,, | 1·70 |
| | 378 | ,, | 1·78 |
| | With red cell contamination | | |
| | 164 | F. | 1·80 |
| | 16 | M. | 2·59 |

The urine from 3 women contained an " activator " of some kind, since on dilution with distilled water the decrease in enzyme activity was greater than on dilution with boiled urine (Fig. 2). In each of 4 normal subjects and 2 patients on whom these investigations have been repeated, the inhibition or activation originally demonstrated remained unchanged.

(c) *Urinary β-glucuronidase activity during the menstrual cycle*

An attempt was made to correlate the enzyme activity with the phases in the normal menstrual cycle. Four normal women between the ages of 20–30 years

FIG. 2.—Activation of urinary β-glucuronidase by substances present in some women's urine.

○ Diluted with distilled water.
× Diluted with boiled urine.

FIG. 3.—Urinary enzyme activity throughout the menstrual cycle in four young women. For each individual the lower graph represents the activity per ml. urine and the upper graph represents the total daily enzyme output.

▬ Menstruation.
—— c —— Contaminated by menstrual blood.
⋮ Time of ovulation.

collected 24-hour specimens throughout one cycle and noted the time of ovulation by recording waking temperatures. Since the cycles were normal it was assumed that peak oestrogen output occurred at about ovulation time and just before menstruation (Pedersen-Bjergaard and Tønnesen, 1948). The urinary β-glucuronidase activity appeared to be unrelated to the phases of the menstrual cycle except that in specimens grossly contaminated with menstrual blood and tissue the activity was elevated (Fig. 3).

TABLE III.—*The β-glucuronidase Activity in Infected Urine where the pH is Within Normal Limits*

| Patient No. | pH | β-glucuronidase activity (units/ml.) | Organism |
|---|---|---|---|
| 299 | 5·2 | 1·10 | Tubercle bacillus. |
| 174 | 5·2 | 1·77 | Mixed coliforms. |
| 148 | 5·3 | 0·21 | Coliforms and *B. proteus.* |
| 150 | 5·6 | 0·66 | Coliforms. |
| 122 | 5·6 | 0·84 | Coliforms and staphylococci. |
| 109 | 5·6 | 1·50 | *B. proteus.* |
| 112 | 5·7 | 0·96 | *E. coli.* |
| 244 | 6·0 | 0·99 | Mixed coliforms. |
| 130 | 6·0 | 1·29 | *E. coli.* |
| 110 | 6·0 | 1·51 | *Ps. pyocyanea.* |
| 161 | 6·0 | 1·51 | *Ps. pyocyanea.* |
| 230 | 6·0 | 1·51 | *E. coli.* |
| 79 | 6·0 | 1·70 | *E. coli.* |
| 194 | 6·2 | 0·3 | *E. coli.* |
| 240 | 6·2 | 0·63 | *E. coli.* |
| 83 | 6·2 | 0·93 | Mixed with *E. coli.* |
| 256 | 6·2 | 1·05 | *Aerobacter aerogenes. Ps. pyocyanea.* |
| 120 | 6·2 | 1·08 | *Aerobacter aerogenes.* |
| 242 | 6·2 | 1·11 | Mixed coliforms. |
| 218 | 6·3 | 1·10 | Mixed coliforms and *Staphylococcus aureus.* |
| 274 | 6·4 | 0·51 | *E. coli.* |
| 58 | 6·4 | 0·66 | Mixed. |
| 253 | 6·4 | 0·90 | *E. coli.* |
| 226 | 6·4 | 1·41 | *Aerobacter aerogenes.* |
| 301 | 6·58 | 0·90 | *E. coli.* |
| 246 | 6·6 | 1·60 | Coliforms. |
| 250 | 6·8 | 0·81 | Coliforms and Enterococci. |

(d) *The effect of alkaline conditions of the urine upon the β-glucuronidase activity* (Tables III and IV)

With slight urinary infection where the pH was not raised above pH 7·0 the enzyme activity was not significantly altered (Table III). In 5 patients with cancer of the bladder the urine was grossly infected and in 4 cases the pH was raised to a value of 9·0 or more. In these patients the urinary enzyme activity was increased. In patients suffering from other diseases where the pH of the urine was abnormally high, in several cases (6 out of 20), the enzyme activity was elevated (Table IV). The effect of incubating normal urine with *B. proteus* or *E. coli* before the assay was variable, the enzyme activity being sometimes increased and sometimes decreased. When the pH of normal urine was increased by the addition of alkali the enzyme activity fell.

TABLE IV.—*Urinary β-glucuronidase Activity in Patients with Urine of High pH*

| Disease | Patient No. | pH | β-glucuronidase activity: Units/ml. urine | β-glucuronidase activity: Units/day | Organism |
|---|---|---|---|---|---|
| Cancer of the bladder | 296 | 7·14 | 4·95 | 3,960 | — |
| | 287 | 9·0 | 2·17 | 4,180 | Enterococci. |
| | 213 | 9·0 | 2·40 | 3,820 | — |
| | 278 | 9·2 | 2·77 | 11,400 | *B. proteus.* |
| | 305 | >9·0 | 3·28 | 3,180 | — |
| Any other disease | 222 | 7·2 | 0·93 | 1,311 | *B. proteus.* |
| | 169 | 7·28 | 0·69 | 2,640 | Sterile. |
| | 86 | 7·4 | 1·14 | 2,040 | Sterile. |
| | 81 | 7·5 | 2·30 | 1,840 | — |
| | 63 | 7·6 | 1·70 | 3,570 | *Aerobacter aerogenes.* |
| | 160 | 8·0 | 2·19 | 4,420 | Non-haemolytic streptococci. |
| | 295 | >8·0 | 0 | 0 | Coliforms + *Streptococcus faecalis.* |
| | 108 | >8·0 | 0·81 | 2,090 | Mixed coliforms. |
| | 50 | >8·0 | 1·18 | 2,840 | Coliform. |
| | 227 | >8·0 | 2·04 | 4,896 | — |
| | 140 | 8·5 | 0·72 | 453 | *E. coli.* |
| | 105 | 9·0 | 1·68 | 2,140 | *B. proteus.* |
| | 198 | 9·0 | 7·65 | 33,600 | *Streptococcus faecalis* and *B. proteus.* |
| | 64 | >9·0 | 0·72 | 630 | Diphtheroid. |
| | 248 | >9·0 | 1·14 | 1,530 | *Aerobacter aerogenes.* |
| | 26 | >9·0 | 1·57 | 2,155 | *B. proteus.* |
| | 89 | >9·0 | 2·93 | 1,570 | *B. proteus.* |
| | 149 | >9·0 | 3·96 | 10,300 | — |
| | 54 | >9·0 | 4·20 | 11,050 | — |
| | 112 | >9·0 | 9·45 | 5,760 | — |

(e) *Urinary β-glucuronidase activity after operation* (Table V)

It was found that within 8 days after major operations performed on parts of the alimentary canal the enzyme activity was significantly increased, which is in agreement with the observation of Boyland and Williams (1956). On the other hand minor operations upon skin or fairly superficial muscle did not result in an elevation of the enzyme activity. The effect of operations on the genito-urinary tract upon the enzyme activity was variable.

TABLE V.—*Urinary β-glucuronidase Activity within* 8 *Days of Operation*

| | | Urinary β-glucuronidase activity | | | | | | | | | |
|---|---|---|---|---|---|---|---|---|---|---|---|
| | | Units/ml. urine | | | "*t*" test against normal subjects | | Units/day | | | "*t*" test against normal subjects | |
| Type of surgery | n | Mean | S.D. | S.E. | *t* | P | Mean | S.D. | S.E. | *t* | P |
| Minor | 20 | 1·19 | 0·39 | 0·088 | 0·98 | Between 0·4 and 0·3 | 1523 | 569 | 127 | 0·52 | 0·6 |
| Major | 13 | 3·72 | 1·68 | 0·466 | 8·08 | Less than 0·01* | 3534 | 1750 | 486 | 5·37 | Less than 0·01* |

S.E. = Standard error of the mean.
* Significantly different.

*β-glucuronidase activity in normal subjects, in cancer of the bladder and in other diseases* (Table VI)

*Group* 1.—In normal subjects the mean activity/ml. urine was 1·05 units with a standard error of the mean of 0·098 and the mean daily excretion was 1405 units/day with a standard error of 160. Boyland *et al.* (1957) found the normal range to be 0·05–1·2 units/ml. and when the daily outputs were calculated from their published figures the mean was 1304 units/day with a range of 162–4890 units.

TABLE VI.—*Urinary β-glucuronidase Activity in Normal Subjects and in Patients with Different Diseases*

| Group | Average age (years) | Units/ml. n | Units/ml. Mean | Units/ml. S.E. | Units/ml. S.D. | "t" test against normal subjects t | "t" test against normal subjects P | Units/day Mean | Units/day S.E. | Units/day S.D. | "t" test against normal subjects t | "t" test against normal subjects P |
|---|---|---|---|---|---|---|---|---|---|---|---|---|
| 1. Normal subjects | 33 | 32 | 1·05 | 0·098 | 0·55 | — | — | 1405 | 160 | 893 | — | — |
| 2. G.U. diseases (total age range) | 44 | 41 | 1·21 | 0·122 | 0·78 | 0·98 | Between 0·4 and 0·3 | 2353 | 270 | 1707 | 2·8 | 0·01* |
| 2a. G.U. diseases (over 50 years of age) | 59 | 21 | 1·31 | 0·227 | 1·04 | 1·14 | Between 0·3 and 0·2 | 2251 | 441 | 2023 | 2·06 | Between 0·05 and 0·02*. |
| 3. "Miscellaneous" diseases (total age range) | 51 | 55 | 1·16 | 0·084 | 0·62 | 0·82 | Between 0·5 and 0·4 | 1719 | 141 | 1047 | 1·4 | Between 0·2 and 0·1. |
| 3a. "Miscellaneous" diseases (over 50 years of age) | 60 | 34 | 1·35 | 0·121 | 0·70 | 1·91 | Between 0·1 and 0·05 | 1649 | 163 | 950 | 1·02 | Between 0·3 and 0·2. |
| 3b. Fractures . . . | 43 | 27 | 2·55 | 0·213 | 1·24 | 6·14 | 0·01* | 2990 | 321 | 1697 | 4·55 | Less then 0·001*. |
| 4. Cancer of the bladder . | 63 | 71 | 1·18 | 0·068 | 0·58 | 1·073 | Between 0·3 and 0·2 | 1831 | 100 | 831 | 2·3 | 0·025* |

S.E. = Standard error of the mean.
* Significantly different.

TABLE VII.—*Daily Volume of Urine Excreted by Normal Subjects and Patients with Different Diseases*

| Group | Volume of urine excreted (ml.) Mean | S.E. | S.D. | "t" test against normal subjects t | P |
|---|---|---|---|---|---|
| 1. Normal subjects . . . . | 1419 | 110 | 577 | — | — |
| 2. Genito-urinary diseases (total age range) | 2094 | 137 | 901 | 3·52 | Less than 0·001*. |
| 2a. Genito-urinary diseases (over 50 years of age) | 1824 | 165 | 755 | 2·108 | Between 0·05 and 0·02*. |
| 3. "Miscellaneous" diseases (total age range) | 1462 | 87 | 635 | 0·356 | Between 0·8 and 0·7. |
| 3a. "Miscellaneous" diseases (over 50 years of age) | 1360 | 34 | 625 | 0·379 | Between 0·8 and 0·7. |
| 3b. Fractures . . . . . . | 1173 | 108 | 571 | 1·529 | Between 0·2 and 0·1. |
| 4. Cancer of the bladder . . . | 1739 | 68 | 601 | 2·408 | Between 0·02 and 0·01*. |

* Significantly different.

TABLE VIII

(*a*) Comparison of the 24-hour enzyme activity in subjects from the normal and " miscellaneous " groups who had excreted over 1700 ml. urine, with the activity in patients with carcinoma of the bladder and with other genito-urinary diseases.

| | | | | " *t* " tests: Urine volumes: Against carcinoma bladder group | | Urine volumes: Against other g.u. diseases | | 24-hour enzyme activity: Against carcinoma bladder group | | 24-hour enzyme activity: Against other g.u. diseases | |
|---|---|---|---|---|---|---|---|---|---|---|---|
| Group | n | Mean volume urine excreted (ml.) | Mean daily enzyme activity (units) | *t* | *P* | *t* | *P* | *t* | *P* | *t* | *P* |
| 1+3>1700 | 19 | 2150 | 2355 | 2·78 | <0·01* | 0·253 | 0·80 | 2·37 | 0·02* | 0·005 | >0·9 |

(*b*) Comparison of the 24-hour enzyme activity in patients with carcinoma of the bladder and other genito-urinary diseases who had excreted less than 1900 ml. urine, with the activity in normal subjects.

| | | | | " *t* " test against normal subjects: Urine volumes | | 24-hour enzyme activity | |
|---|---|---|---|---|---|---|---|
| Group | n | Mean volume urine excreted (ml.) | Mean enzyme activity (units) | *t* | *P* | *t* | *P* |
| 2+4<1900 | 55 | 1323 | 1687 | 0·899 | Between 0·4 and 0·3 | 1·453 | Between 0·2 and 0·1 |

* Significantly different.

*Groups* 2, 3 *and* 4.—The results in this section are from urine within the normal pH range and not contaminated by excess red cells. During the urine collection the patients were not suffering from pyrexia, had not undergone operations within the previous fortnight and were not having steroid hormone therapy.

In the original " miscellaneous " group it became obvious that patients with fractures had an enzyme activity higher than any other. These cases were therefore removed and considered separately.

Apart from this (Group 3b) the β-glucuronidase activity per ml. urine in any of the groups (2, 2a, 3, 3a, or 4) was not significantly different from that in normal subjects.

The daily enzyme output in both the total " miscellaneous " group (3) and its sub-group of patients over 50 years of age was also not significantly different from the normal value. The enzyme output per day, however, in the total " other genito-urinary diseases "(Group 2) and in its sub-group was significantly increased as it was also in the group of cancer of the bladder cases (4).

It was suspected that the increased enzyme output per day in both groups 2 and 4 might be a mere statistical effect of the significantly larger volumes of urine excreted (Table VII) as a result of the greater fluid intake encouraged in these two groups of patients. Since it was very difficult to obtain reliable information about the fluid intake the following, admittedly arbitrary, method was used to test such a hypothesis. The daily enzyme output values of individuals in the normal and " miscellaneous " groups who had excreted a volume of urine of 1700 ml. or more Group (1 + 3 > 1700), were statistically compared with the enzyme activity in Groups 2 and 4 (Table VIII). No significant difference was found between the mean urine volumes in this ($1 + 3 > 1700$) group and the other genito-urinary diseases (2), nor was there any significant difference in the daily enzyme excretion. It was even found that the mean urine volume of group ($1 + 3 > 1700$) was significantly higher than the mean volume of the carcinoma of the bladder group (4), and, correspondingly, the daily enzyme activity in groups ($1 + 3 > 1700$) was higher than in the cancer of the bladder cases.

As an added check, all results from patients with carcinoma of the bladder and other genito-urinary diseases who had excreted a daily volume of less than 1900 ml. (again an arbitrary figure), were combined (group ($2 + 4 < 1900$)) and compared with the enzyme activities in normal subjects (1). There was no significant difference in the enzyme output in these two groups ; the mean volumes of urine excreted were also not significantly different.

The results from Groups 1, 2a, 3a and 4 indicated that, although the mean values were higher in the older groups, age had no significant effect on the urinary $\beta$-glucuronidase activity/ml. urine. In both the sub-groups of patients over 50 years of age the mean volumes of urine excreted were less, but not significantly, than in the corresponding main group, and the mean daily enzyme activities were also lower.

In the fracture group, 3b, both the activity per ml. of urine and the daily enzyme output were significantly raised compared with the normal values for at least 10 days after the fracture (Lewis and Plaice, 1959). As the mean volume of urine excreted was lower than normal it was assumed that this was in fact a true difference.

### DISCUSSION

The enzyme concentration per ml. of urine is an indication, at any particular time, of the possible hydrolytic opportunities, but the length of time the bladder mucosa is exposed to the carcinogens is another factor which must play a part.

The enzyme activity per ml. of urine was not significantly increased in the cancer of the bladder patients, which is not in accord with the results of Boyland *et al.* (1955). When, however, the enzyme activity per day was considered, both these and the other urinary tract diseases had significantly increased activity compared with the normal value. This increase was not only not specific for the cancer of the bladder patients but it appeared to be a statistical consequence of the greater volumes excreted. This apparent relationship between the volume excreted and the daily enzyme output is difficult to understand.

Boyland and his colleagues (1955) found no $\beta$-glucuronidase inhibitors in the urine whereas in the present series 86 per cent of the samples from men and 52 per cent from women had an inhibitor present. The presence of an endogenous $\beta$-glucuronidase inhibitor in many types of tissue and in serum is well known (Walker and Levvy, 1953) and an enzyme inhibitor in urine has been demonstrated (Abul-Fadl, 1957). Levvy (1956) points out that an important cause of inhibition of hydrolysis of a particular glucuronide is the presence in urine of other glucuronide conjugates. This will in any case introduce errors of unknown magnitude in the determination of urinary $\beta$-glucuronidase activity. It is of interest to note that under the experimental conditions used in this study, the urine of some women contained no inhibitors and appeared to have " activators ". If $\beta$-glucuronidase plays an important rôle in releasing bladder carcinogens it is therefore difficult to understand why women have a lower incidence of bladder cancer than men unless there is some difference between the sexes in the length of time that the carcinogen can act upon the bladder epithelium.

The method used in this work and in the investigation of Boyland *et al.* (1955) entails a dilution of urine with acetate buffer and substrate. This dilution of any inhibitor or " activator " does not give an accurate picture of the enzyme activity in the bladder. Walker and Levvy (1953) discussing an endogenous inhibitor affecting rat-liver $\beta$-glucuronidase activity in *in vitro* investigations state that it is by no means certain that the inhibitor has any effect *in vivo*. To study the enzyme in the liver they homogenised the tissue, thus disrupting the cell constituents and perhaps causing contact of enzyme and inhibitor which would not naturally occur. In urine however, it is more likely that the inhibitor is as active in the bladder as it is *in vitro*. The enzyme activity in the bladder is usually less than that estimated *in vitro* at an optimum pH of 4·5. It is possible that the inhibitor or activator action differs at the pH of the urine in the bladder from that under the experimental conditions (Smith and Mills, 1953). Odell and Fishman (1950), investigating the $\beta$-glucuronidase activity in human endometrium, found that there was a variation of activity during the menstrual cycle. They found that the lowest values of the enzyme are immediately preceding and following the period of the menses and the highest values occur in the interval between. It seemed possible that these changes in endometrial activity might be reflected in the urinary output although Fishman *et al.* (1951) found no consistent correlation between the blood enzyme activity and the phases of the cycle. The function of $\beta$-glucuronidase is still obscure. Not only is it possible that the enzyme might be involved in the synthesis of oestrogen and pregnanediol glucuronides, although this is now considered unlikely, but it might also be involved in structural proliferation of the uterus in the phase before ovulation and in the production of mucin in the secretory phase. In women, cancer of the bladder usually occurs in the post-menopausal stage and the enzyme determinations would not be affected by any cyclical changes. It was thought at the beginning of this investigation that in the control groups, where there was a greater number of younger women, the menstrual cycle might have affected the enzyme activity. If, however, $\beta$-glucuronidase plays a rôle in any of the metabolic processes during the menstrual cycle it was not evident in the urinary activity of the small series investigated.

With regard to the increased urinary enzyme activity found after some operations and apparently associated with fairly large amounts of tissue proliferation, Levvy's review (1956) must be borne in mind in which he emphasises that there are many results obtained by different workers which do not support the generalisation that $\beta$-glucuronidase activity is invariably related to tissue growth. Although this might be the reason for the elevation during the healing of fractures there may be a relationship between hydrolysis of chondroitin and other mucopolysaccharides and the enzyme activity, as suggested by Levvy (1956).

It is possible that the discrepancy between the results of Boyland *et al.* (1955) and those reported here is due to a difference in the stage of the disease when the patient was investigated. The patients investigated at Southmead Hospital (except for three of the cases in Table IV) were fairly fit and were able to resume normal activities upon discharge after review cystoscopy. In advanced, inoperable cases, even where the pH of the urine is normal, it is feasible that contamination of the urine from the tumour tissue which, Boyland and his colleagues (1955)

have shown to contain high enzyme activity, would cause an increased urinary activity. Our results on the enzyme activity of the urinary sediments from patients with cancer present in the bladder were inconclusive. On the other hand, Boyland's cases may also have had significant bony metastases, another differential feature from ours. It was considered possible that as new bone formation was associated with an increase in the urinary glucuronidase activity, some forms of metastases in bone might have a similar effect. To date a small series of results in patients with such metastases (unpublished) has not been helpful, possibly on account of the steroid hormone therapy given.

Since this was written our attention has been drawn to two recent papers concerned with urinary $\beta$-glucuronidase activity in patients with cancer of the bladder. Ohkubo, Sonoda and Kusonoki (1958) in an investigation of the enzyme values of serum, urine and tissue of patients with urological diseases found that in 6 out of 7 cases of bladder cancer the urinary enzyme values were higher than in 16 normal subjects. Mattea and Pietra (1959) observed that in 30 subjects with industrial cancer of the bladder 29 had increased enzyme activity in urine diluted ten times compared with the activity of similarly diluted urine of 4 normal subjects.

The absolute values obtained by the authors of these two papers cannot be directly compared with each other, with the results of Boyland *et al.* (1957) or with the results in the present paper, since experimental factors such as urine and substrate concentration differed.

### SUMMARY

1. Urinary $\beta$-glucuronidase activity was estimated in patients with cancer of the bladder and in a large number of controls, including patients with other diseases, in addition to normal subjects.

2. $\beta$-glucuronidase inhibitors were found in 86 per cent of the men and 52 per cent of the women investigated for this effect.

3. The enzyme activity per ml. urine as well as the daily output were both significantly higher than normal in some cases after major surgery and also, for at least 10 days after fractures.

4. Apart from this the enzyme activity per ml. urine was not significantly different in cancer of the bladder from that in normal subjects or in any of the diseases investigated.

5. The 24-hour output of the enzyme was significantly increased in cancer of the bladder and in other genito-urinary diseases. Data are presented which appear to correlate this increase with the increased volume of urine excreted by such patients.

This work was supported by a grant from the British Empire Cancer Campaign (to C. H. J. P.). We wish to thank Mr. Ashton Miller who inspired this work, Mr. Mitchell and Mr. Slade for their kindness and help in putting this material at our disposal. We also would like to thank Professor Gordon Lennon, University Department of Obstetrics and Gynaecology, Southmead Hospital, for provision of laboratory space. We are greatly indebted to Dr. G. Herdan, Statistician, Department of Preventive Medicine, the University of Bristol, for his valuable help with the statistical analysis. We also wish to thank the nursing and ancillary staff of the hospitals concerned for their co-operation.

### REFERENCES

Abul-Fadl, M. A. M.—(1957) *J. clin. Path.*, **10**, 387.
Allen, M. J., Boyland, E., Dukes, C. E., Horning, E. S. and Watson, J. G.—(1957) *Brit. J. Cancer*, **11**, 212.
Bonser, G. M., Bradshaw, L., Clayson, D. B. and Jull, J. W.—(1956) *Ibid.*, **10**, 539.
Booth, J., Boyland, E. and Manson, D.—(1955) *Biochem. J.*, **60**, 62.
Boyland, E., Gasson, J. E. and Williams, D. C.—(1957) *Brit. J. Cancer*, **11**, 120.
*Idem*, Wallace, D. M. and Williams, D. C.—(1955) *Ibid.*, **9**, 62.
*Idem* and Williams, D. C.—(1956) *Rep. Brit. Emp. Cancer Campgn.*, **34**, 40.
Fishman, W. H., Kasdon, S. C., Bonner, C. D., Fishman, L. W. and Homburger, F.—(1951) *J. clin. Endocrin.*, **11**, 1425.
*Idem*, Springer, B. and Brunetti, R.—(1948) *J. biol. Chem.*, **173**, 449.
Hueper, W. C., Wiley, F. H. and Wolfe, H. D.—(1938) *J. industr. Hyg.*, **20**, 46.
*Idem* and Wolfe, H. D.—(1937) *Amer. J. Path.*, **13**, 656.
Levvy, G. A.—(1956) ' Vitamins and Hormones ', **14**, 289 (edited Harris, R. S., Marrian, G. F. and Thimann, K. V.). New York (Academic Press Inc.).
Lewis, F. J. W. and Plaice, C. H. J.—(1959) *Nature*, **184**, 1249.
Mattea, E. and Pietra, E.—(1959) *Tumori*, **45**, 86.
Odell, L. D. and Fishman, W. H.—(1950) *Amer. J. Obstet. Gynec.*, **59**, 200.
Ohkubo, T., Sonoda, T. and Kusunoki, T.—(1958) *Urol. int.*, **7**, 167.
Pedersen-Bjergaard, K. and Tønnesen, M.—(1948) *Acta endocrin.*, **1**, 38.
Smith, E. E. B. and Mills, G. T.—(1953) *Biochem. J.*, **54**, 164.
Talalay, P., Fishman, W. H. and Huggins, C.—(1946) *J. biol. Chem.*, **166**, 757.
Walker, P. G. and Levvy, G. A.—(1953) *Biochem. J.*, **54**, 56.
Wallace, D. M.—(1959) ' Neoplastic Diseases at Various Sites ', 2, ' Tumours of the Bladder '. Edinburgh and London (E. S. Livingstone, Ltd.).